# 160 Empowering future healthcare leaders and clinical researchers across a decade: UCLA’s Clinical and Translational Science Institute – Research Associates Program – CORRIGENDUM

**DOI:** 10.1017/cts.2025.75

**Published:** 2025-04-28

**Authors:** Omar Selim, Tiffany Chen, Laurie Shaker-Irwin, Noah Federman, Jim Morrison, Denise Gellene, Angshuman Saha, Brisa Garcia

The above abstract published with a missing author. Omar Selim was missing from the author list.

This has been updated in the original abstract.
